# Identification and Utilization of Donor and Recipient Genetic Variants to Predict Survival After HCT: Are We Ready for Primetime?

**DOI:** 10.1007/s11899-014-0246-x

**Published:** 2015-02-21

**Authors:** Lara E. Sucheston-Campbell, Alyssa Clay, Philip L. McCarthy, Qianqian Zhu, Leah Preus, Marcelo Pasquini, Kenan Onel, Theresa Hahn

**Affiliations:** 1 Department of Cancer Prevention and Control, Roswell Park Cancer Institute, Elm and Carlton Streets, Buffalo, NY 14263 USA; 2 Department of Medicine, Roswell Park Cancer Institute, Elm and Carlton Streets, Buffalo, NY 14263 USA; 3 Department of Biostatistics, SUNY-Buffalo, Buffalo, NY USA; 4 Center for International Blood and Marrow Transplant Research, Medical College of Wisconsin, Milwaukee, WI USA; 5 Department of Pediatrics, The University of Chicago, Chicago, IL USA

**Keywords:** Hematopoietic cell transplantation (HCT), HLA typing, Infection prophylaxis, Non-HLA biologic predictors, SNPs

## Abstract

Overall survival following hematopoietic cell transplantation (HCT) has improved over the past two decades through better patient selection and advances in HLA typing, supportive care, and infection prophylaxis. Nonetheless, mortality rates are still unsatisfactory and transplant-related mortality remains a major cause of death after unrelated allogeneic HCT. Since there are no known pre-HCT, non-HLA biologic predictors of survival following transplant, for over a decade, scientists have been investigating the role of non-HLA germline genetic variation in survival and treatment-related mortality after HCT. Variation in single nucleotide polymorphisms (SNPs) has the potential to impact chemotherapy, radiation, and immune responses, leading to different post-HCT survival outcomes. In this paper, we address the current knowledge of the contribution of genetic variation to survival following HCT and discuss study design and methodology for investigating HCT survival on a genomic scale.

## Introduction

The most successful curative therapy for many malignant hematologic diseases is hematopoietic cell transplantation (HCT). However, the success of this treatment is limited by transplant-related mortality (TRM). Better patient selection and advances in HLA typing combined with supportive care and infection prophylaxis have improved survival over the past two decades [1, 2•, 3]. Nonetheless, TRM remains a major cause of death, with disease-related mortality (DRM) the other largest contributor [4]. Although several clinical variables, including disease status at transplant, stem cell source, graft source, and CMV status are associated with survival outcomes [3], there are currently no established genetic predictors of survival after transplantation outside of *HLA* matching. Variation in single nucleotide polymorphisms (SNPs) may lead to differential gene transcription, translation, and protein structure. These changes have the potential to modify immune responses or side effects of chemotherapy and/or radiation, and thus, survival outcomes in HCT patients [5, 6]. Various candidate genes have been tested for association with survival outcomes. Variants have been selected in genes relating to immune response to infection and inflammatory reactions, with the goal of understanding the biological basis of TRM and DRM and ultimately developing a better understanding of individual risk conveyed by genomic loci outside *HLA*. In studies of allogeneic HCT outcomes, the individual and joint effects of recipient and donor genotypes have been tested with the intent of developing a clinically applicable donor selection strategy to improve transplant success [7–9]. Another approach has focused on selecting and evaluating SNPs in genes within drug metabolism/detoxification pathways. By demonstrating genetic associations with transplant outcomes after exposure to various chemotherapeutic agents or combinations, the potential to assign a conditioning regimen based on genotype becomes a possibility [10, 11]. To date, these candidate gene approaches have not been conclusive, in part due to sample size limitations and marked heterogeneity in population, disease, *HLA* matching, donor, and graft source. The objective of this article is to understand where studies of germline genetic variation have taken the transplant field in the hunt for clinically valid and actionable genetic variation associated with survival after HCT and how using genomics, versus genetics, may lead to better outcomes for patients [12••, 13]. We focus only on the role of germline genetic variation on survival after transplant since other studies have eloquently reviewed SNP associations with outcomes such as susceptibility to leukemia and chemotherapy toxicity [14] and the incidence and severity of graft-versus-host disease (GvHD) [15••]. We address current knowledge about the contribution of germline SNPs to survival following allogeneic HCT, first reviewing the published evidence for a role of non-*HLA* genetics in survival following transplant irrespective of, and considering, chemotherapeutic exposures. We then consider where these studies are in the translational research continuum and discuss genomic study design and methodological considerations when measuring competing risk outcomes.

## Overview of Associations of Candidate Genes with Survival After HCT

Candidate gene studies have tested SNP associations with cause-specific (TRM and DRM) and overall survival in redox metabolism genes (*GSTM1*, *UGT2B17*) [16, 17] and cytokine and chemokine genes and their receptors (*IL7 receptor-α*, *IL-10* and *TNF-α*, *IL-1*, *IL-1-β*, *IL-1-α*, *IL-6*, *IL-10*, *IL-10R*, *IL-23*, *IL23R*, *CCL2*, *CCR5*, *TLR9*) in both donors and recipients [18–30]. Other studies focused on variants in genes responsible for immune response and recognition including *FCGR3A* [31], *CTLA4* [23, 24, 32–35], LCT [36], and *NOD2/CARD15* [37–49], as well as *VDR* [50–54] and *MTHFR* [54, 55] and *THBD* [56]. Initial results were promising, reviewed in [57], and yielded some significant associations with overall survival (OS) and TRM after related and unrelated donor HCT. However, follow-up studies did not replicate SNP associations with outcomes, either because the initial study population was so small the association was false, the replication was equally small, and/or the follow-up was not rigorously designed for replication [23, 38, 45–47].

### Associations of NOD2/CARD15 with Survival After HCT

An excellent example of this is *NOD2/CARD15*, selected for study in transplant outcomes as SNPs in *NOD2/CARD15* were found to be associated with Crohn’s disease, and there are similarities in this chronic inflammatory disorder of the gastrointestinal tract and GvHD symptoms. Few genes have been studied so extensively in relation to HCT with so many conflicting results, reviewed in [58]. While the initial association with an increased risk of TRM [42] was confirmed by a few studies [40, 44, 48], multiple other studies showed no association with survival following HCT [38, 45, 46, 59], with additional work hinting that perhaps results varied by use of T cell-depleted grafts [43]. The largest *NOD2/CARD15* study to date, 567 donor-recipient pairs both *HLA* matched and mismatched with primary diagnoses including hematologic malignancies, non-hematologic malignancies, and nonmalignant diseases, found only a borderline association (*p* = .049) of a recipient SNP with increased TRM and conflicting results in the non-malignant patient groups [60].

Collectively, these SNP-survival association studies appear to be dependent on the combination of transplant regimen, donor cell source, disease, and HLA matching and even further compounded by the fact that SNP frequencies vary significantly by race and ethnicity [61, 62]. The intrinsic complexity of these results is further obfuscated as estimates of effect size and *p* values were obtained from prohibitively small studies, in most cases with 100–200 patients. In addition, only limited variants in this region were studied for associations with TRM, DRM, or OS. Given the polymorphic nature of *NOD2* and the varying linkage disequilibrium structure by race and ethnicity [58], a more thorough investigation in homogenous appropriately sized populations is needed.

This extensive body of work is an excellent illustration of the consequences of heterogeneity and small sample size on studies of the relationship of germline genetics with survival following HCT. Definitive results from large-scale and replicated genetic studies are an imperative first step to finding clinically valid, and ultimately clinically actionable, variants for incorporation in treatment planning [12••, 63].

## Pharmacogenetic Associations with Survival after HCT

Busulfan (Bu) and cyclophosphamide (Cy), the most commonly used alkylators in high-dose conditioning regimens prior to HCT, are associated with inter-individual variation in both relapse and toxicity [64]. While the unpredictable metabolism of these agents can lead to unintentional overdosing, an alternative explanation for excessive toxicity and differential adverse effects among patients given a Bu/Cy conditioning regimen is genetics [65]. SNPs in the CYP450 and glutathione enzyme families have been shown to affect clinical outcomes, relapse, and drug-related toxicities after HCT. Specifically response to Cy/Bu has been hypothesized to be due to variation in Cy metabolism by *CYP2B6* and *CYP2C19* which activates Cy to 4-hydroxy Cy [66, 67] and metabolizing enzymes involved in Bu conjugation, namely glutathione *S*-transferase (*GST*) isoenzymes A1 (*GSTA1*) and M1 (*GSTM1*). However, as with the studies of survival-SNP associations, pharmacogenetic studies of survival after HCT are plagued with inconsistencies due to small sample sizes compounded by the heterogeneity in exposure (drug) and dose.

### Association of the *CYP* Gene Family with Survival

*CYP2C19* encodes a well-characterized and highly polymorphic enzyme that metabolizes Cy (among other drugs). Individuals can be grouped into poor (PM), intermediate (IM), and extensive (EM) metabolizers by presence or absence of active enzymes. PM (CYP2C19*2/*2, *2/*3, and *3/*3) metabolize drugs more slowly, show prolonged side effects [68, 69], and have a higher rate of TRM, but no association was seen with OS [59]. Although this was one of the larger studies of TRM, the low frequency of PM (approximately 3 % of the study population) necessitated a design with considerably larger sample sizes for the appropriate statistical power to detect the true effect size. Subsequent examinations of *CYP2C19* have again suffered from the tyranny of small numbers. Melanson et al. [70] redefined the *CYP2C19*, PM and IM classification previously used [59] and showed associations of CYP2C19*2/*2 with worse progression-free survival (PFS) and OS but not with non-relapse mortality. This could be due to myeloablative doses of conditioning, the sequence of conditioning regimen drugs, or interactions with other drugs which may diminish or alter the detectable genetic effects. Consistent genetic and phenotype definitions are imperative for replications.

*CYP2B6* is also an important enzyme that helps determine the rate of Cy clearance; however, only suggestive associations with survival following HCT have been shown among individuals who are considered ultra-rapid metabolizers, but no impact was seen on overall survival [70]. As with the *CYP2C19* extensive metabolizer group, the *CYP2B6* variants defining ultra-rapid metabolizers were not common, yielding a small comparison group. These *CYP* studies, like the *NOD2/CARD15* research, highlight the importance, and challenge, in designing appropriately sized discovery and replication studies.

### Association of Glutathione S-transferase (GST) Superfamily with Disease-Free Survival

Bu-containing conditioning regimens show even greater inter-patient variability in efficacy and toxicity than Cy. Intravenous vs oral administration of Bu, as well as differences in GI absorption, can generate variability in pharmacokinetics, drug clearance and drug activity. Therefore, patients receiving myeloablative Bu prior to HCT have therapeutic drug monitoring as standard clinical practice [71]. A high Bu steady-state plasma concentration can be toxic, whereas low concentrations are associated with poor engraftment and higher relapse risk [72]. Consequently, clinical outcomes are improved by targeting plasma concentrations and thus variants in the predominant metabolizing enzymes involved in Bu conjugation, glutathione *S*-transferase (*GST*) isoenzymes A1 (*GSTA1*) and M1 (*GSTM1*), have been tested for association with survival after transplant [10, 17, 54, 66–68, 72–75]. Yee et al. found a SNP in the *GSTM1-GSTM5* locus, rs3754446, associated with an almost twofold shorter disease-free survival in two cohorts of acute myeloid leukemia (AML) patients treated with chemotherapy-based autologous HCT. Despite replicating in both cohorts (*p* = .001 and *p* = .028), the finding was not significant after correction for multiple testing [10]. The authors found similar relationships with rs4148405 in *ABCC3*, although while passing multiple testing correction in cohort 1 (*p* < 10e−06), it did not replicate in cohort 2. Additional genetic association studies have provided some evidence that variants in the genes in the ATP-binding cassette (*ABC*) family are associated with outcome [75, 76].

While other adverse associations with *GSTM* polymorphisms have been shown with transplant-related toxicities [74] and in other cancers, reviewed in [77], the *GSTM1* associations seen by Yee et al. have not replicated [54, 74]. Again this could be due to the population studied (Rocha et al. analyzed associations in HLA-identical sibling donor-recipient pairs, while Hahn et al. analyzed overall survival in groups of autologous and related and unrelated donor allogeneic patients), the small heterogeneous sample sizes, the differences in the exact *GSTM* variation studied, or even subtle population substructure.

It is important to note that while this pharmacogenetic research is inconclusive, these drug metabolism pathways are well established and thus merit further investigation in larger cohorts. This approach has been done previously with success in relation to genetic associations with GvHD following HCT [78].

In 2007, an article in *Blood* was published, highlighting the rationale for a genome-wide approach in studying hematologic etiology and disease outcome [79•], and scientists have eagerly moved in this direction [15••]; however, since this time, only candidate gene studies have been published (Table 1). Of these, only two have replicated significant findings in a second independent population and one result has demonstrated enough validity to pursue clinical application in a multicenter trial currently underway.

**Table 1:** SNP association studies of survival outcomes following HCT since 2007

Article [reference number]	Donor source	Genotyping performed in donors (D), recipients (R), and both (D/R)	Disease§	Gene	Findings	Initial *N*/replication *N*
Jagasia M et al. [24]	alloHCT	D/R	Mixed	CTLA-4	Donor variant ↓RFS, ↓OS	164 D-R pairs/0 D-R pairs
					Recipient variant ↓RFS ↓OS	
Perez-Garcia A et al. [33]	Sibling alloHCT	D/R	Mixed	CTLA-4	Donor variant ↓OS	536 D-R pairs/0 D-R pairs
				CD14		
Vannucchi AM et al. [32]	alloHCT	D/R	Mixed	CTLA-4	NS	147 D-R pairs/0 D-R pairs
				CD14		
Sengsayadeth S et al. [23]	alloHCT	R	AML + MDS	CTLA-4	NS	1463 R/0 R
Wu J et al. [34]	alloHCT	D/R	Mixed	CTLA-4	Donor variant ↓DFS	123 R/0 R
Metaxas Y et al. [35]	alloHCT	D/R	Acute leukemia	CT60	Donor variant ↑DFS	79 D-R pairs/0 D-R pairs
				CT60	Recipient variant ↑DFS	
Rachakonda SP et al. [56]	alloHCT	R	Mixed	THBD	Recipient variant ↑NRM	306 R/321 R
Hauser H et al. [36]	alloHCT	D/R	Mixed	LCT	Donor variant ↑OS ↓TRM	111 D-R pairs/0 D-R pairs
					Donor variant ↑TRM	
Kim DD et al. [37]	alloHCT	D/R	Mixed	IL2, IL6R, FAS, EDN1, TGFB1 NFKBIA	Donor NOS1 variant ↓OS	307 D-R pairs/87 D-R pairs
					Donor NOD2/CARD15 and recipient IL6R variants ↓RFS	
				NOS1, IL1B, TGFB2, NOD2/CARD15, TNFRII, IL1R1, FCGR2A	Donor NOS1 variant ↑NRM	
Carvalho A et al. [26]	alloHCT	D/R	Mixed	IL17A	Recipient IL23R variant ↑OS	201 D-R pairs/0 D-R pairs
				IL17F		
				IL23R		
Ambruzova Z et al. [27]	alloHCT	D/R	Mixed	IL6	Donor IL6 variant ↓OS	121 D-R pairs/45 D-R pairs
				CCL2	Donor to recipient CCL2 variants ↓OS and ↑TRM	
Kim M et al. [25]	autoHCT and alloHCT	R	AML	FLT3	Recipient IL10 variant ↓OS	43 R/0 R
				IL10		
Tseng LH et al. [28]	alloHCT	R	Mixed	IL10	NS	936 R/0 R
				IL10R		
Dickinson AM et al. [51]	Sibling alloHCT	D/R	CML	IL1RN	Donor TNFRSFIB (negative) variant and donor IL10 (negative) variant and donor IL1RN (positive) variant ↑TRM ↓OS	228 D-R pairs/0 D-R pairs
				IL4		
				IL6		
				IL10		
				IFNG		
				ESR1		
				VDR		
				TNFRSFIB		
				TNF		
Mehta PA et al. [22]	alloHCT	D/R	CML	IL1A	NS	426 D-R pairs/0 D-R pairs
Elmaagacli AH et al. [29]	Sibling alloHCT	D/R	AML	TLR9	Recipient TLR9 variant ↑OS ↓TRM	142 D-R pairs/0 D-R pairs
				IL23R		
				NOD2		
Mayor NP et al. [44]	alloHCT	D/R	Acute leukemia	NOD2/CARD15	Donor variants ↓OS ↑relapse	192 D-R pairs/0 D-R pairs
Granell M et al. [40]	sibling alloHCT	D/R	Mixed	NOD2/CARD15	Donor-recipient variants ↓DFS	71 D & 85 R/0 D and R
Nguyen Y et al. [45]	alloHCT	D/R	Mixed	NOD2/CARD15	NS	390 D-R pairs/0 D-R pairs
Kreyenberg H et al. [60]	alloHCT	D/R	Mixed	NOD2/CARD15	Donor-recipient ↑TRM	567 D-R pairs/0 D-R pairs
					Donor-recipient variants ↓OS	
van der Velden WJ et al. [48]	Sibling alloHCT	D/R	Mixed	NOD2	Recipient variants ↑TRM	85 D-R pairs/0 D-R pairs
					Donor-recipient variants ↑TRM	
Holler at al.[43]	alloHCT	D/R	Mixed	NOD2/CARD15	Donor variant ↑TRM	342 D-R pairs/0 D-R pairs
Holler E et al. [49]	alloHCT	D/R	Acute leukemia	NOD2/CARD15	Donor-recipient variants ↑TRM ↓OS	358 D-R pairs/0 D-R pairs
Sairafi D et al. [46]	alloHCT	D/R	Mixed	NOD2/CARD15	NS	198 D-R pairs/0 D-R pairs
Van der Straaten HM et al. [38]	alloHCT	D/R	Mixed	NOD2/CARD15	NS	192 D-R pairs/0 D-R pairs
McDermott DH et al. [30]	alloHCT	D/R	Mixed	CCR5	Recipient variants ↑DFS ↑OS	1370 D-R pairs/0 D-R pairs
					Donor-recipient variants ↑DFS ↑OS	
Cho HJ et al. [50]	Sibling alloHCT	R	Mixed	VDR	Recipient variants ↑OS and ↑DFS	147 R/0 R
					Recipient variants ↑OS and ↑DFS	
Bogunia-Kubik K et al. [52]	Sibling alloHCT	D/R	Mixed	VDR	Recipient variants ↑death	123 D-R pairs/0 D-R pairs
Takami A et al. [31]	alloHCT	D/R	Mixed	FCGR3A	Recipient variant ↑OS ↑TRM	99 D-R pairs/0 D-R pairs
KIR						
Cooley S et al. [87]	alloHCT	D	Acute leukemia	KIR	Donor variants ↑DFS	1409 D-R pairs/0 D-R pairs
					Donor variants ↓DFS	
Cooley S et al. [85]	alloHCT	D/R	AML	KIR	NS	1532 D-R pairs/0 D-R pairs
				HLA-C	Donor KIR variants with recipient HLA-C variants ↑LFS	
Cooley S et al. [88]	alloHCT	D/R	AML	KIR	Donor variants ↑OS	448 D- R pairs/0 D-R pairs
					Donor variants ↑RFS	
Venstrom JM et al. [80]	alloHCT	D/R	AML	KIR2DS1		1277 D-R pairs/0 D-R pairs
				KIR3DS1		
				HLA-C1	NS	
				HLA-C2	Recipient with donor KIR variants ↓mortality	
Bari R et al. [84]	alloHCT	D/R	Acute leukemia	KIR	Donor KIR variant ↑OS ↓DP	313 D-R pairs/0 D-R pairs
				HLA	Donor-recipient mismatched KIR+ with HLA-C variants ↑OS and ↓LP	
Weisdorf D et al. [86]	alloHCT	D/R	AML	KIR	Donor-recipient mismatched KIR and HLA variants ↑OS	24 D-R pairs/0 D-R pairs
				HLA		
Gagne K et al. [8]	alloHCT	R	Mixed	KIR	Donor-recipient mismatched KIR variants ↓OS	264 R/0 R
				HLA		
Pharmacogenomics						
Elmaagacli AH et al. [59]	alloHCT	D/R	Mixed	CYP2C19	Recipient variants ↑TRM	289 D-R pairs/0 D-R pairs
Melanson SE et al. [70]	alloHCT	R	Mixed	CYP2B6	Recipient variant ↓PFS	359 R/0 R
				CYP2C19	Recipient variant ↓PFS ↓OS	
Koh Y et al. [11]	related alloHCT	D/R	AML	CYP3A5	NS	156 D-R pairs/0 D-R pairs
				MDR1	Recipient variant ↑OS ↓TRM	
Rocha V et al. [54]	alloHCT	D/R	Mixed	CYP2B6	NS	107 D-R pairs/0 D-R pairs
				GST	NS	
				VDR	Recipient variant ↑TRM and ↑OS	
				MTHFR	NS	
Hahn T et al. [74]	autoHCT and alloHCT	R	Mixed	GSTM1	NS	321 R/0 R
				GSTT1		
Bonifazi F et al. [73]	alloHCT	R	Mixed	GSTA2	Recipient variants ↑TRM ↓OS	185 R/0 R
Yee SW et al. [10]	autoHCT	R	AML	ABCC3	Recipient variant ↓DFS	154 R/125 R
				GSTM1-GSTM5	Recipient variants ↓DFS	
Wang F et al. [76]	autoHCT and alloHCT	R	Acute leukemia	ABCG2	Recipient variant ↑DFS ↑OS	184 R/0 R
					Recipient variant ↓DFS ↓OS	
Kim I et al. [55]	Sibling alloHCT	R	Mixed	MTHFR	Recipient variants ↓TRM	72 R/0 R

*OS* overall survival, *RFS* relapse-free survival, *PFS* progression-free survival, *DFS* disease-free survival, *LFS* leukemia-free survival, *TRM* treatment-related mortality, *NRM* non-relapse mortality, *NS* non-significant, *D* donors, *R* recipients

### Independent and Joint Effects of KIR and HLA-C in AML Patients Treated with alloHCT

The polymorphic killer-cell immunoglobulin-like receptors (KIRs) recognize *KIR* ligands. HLA molecules activate *KIR* receptors at the cell surface and therefore are considered *KIR-*ligands. The *KIR* ligands are grouped into three major categories based on the amino acid sequence determining the KIR-binding epitope in HLA-C and HLA-B molecules. All expressed *HLA-C* alleles are of the C1 or C2 group and most *HLA-B* alleles can be classified as either Bw4 or Bw6 [80, 81]. This *HLA-KIR* interaction was first explored in *HLA* haplo-identical transplantation [73] and later investigated in other allogeneic donor settings in hopes it may stimulate GVL reactions in HCT [81–84]. More recently, survival outcomes under varying combinations of donor genetic variation in *KIR* and recipient HLA class I have been tested [84–89••]. These studies focused on AML outcomes after an unrelated donor HCT (URD-HCT) in pediatric and adult patients and have shown similar results in both the direction and magnitude of the impact of the KIR donor genotype on survival outcomes. Therefore, a prospective clinical trial incorporating *KIR* genotyping into URD selection for AML is currently accruing patients (http://www.clinicaltrials.gov/,NCT01288222). This is the first clinical trial of HCT outcomes involving non-*HLA* genetics and is the result of consecutively larger studies rigorously demonstrating a genetic association in homogenous study populations.

*KIR* can be broadly categorized into two haplotypes [90–92]: the A haplotype, with at most a single activating gene, *KIR2DS4*, and the B haplotype, with *KIR2DS4* plus at least one of the following activating genes: *KIR3DS1*, *KIR2DS2*, *KIRDS3*, or *KIRDS5*. Individuals who are *B/x* (either *B/B* or heterozygous *B/A*) are described as having an activating *KIR* genotype. The first study to demonstrate an association of recipient outcome with *KIR* activating genes included 209 HLA-matched and 239 HLA-mismatched T-replete URD-HCT for AML [88]. Three-year overall survival was significantly higher after transplantation from a *KIR* B/x donor afforded the recipients better overall and relapse-free survival when compared with A/A donors. The protective effect of donor *B/x* genotype was replicated in a larger cohort of early, intermediate, and advanced AML undergoing T-replete URD-HCT (*n* = 1086). However this advantage was not seen in acute lymphoblastic leukemia (ALL) patients (*n* = 323).

### HLA-KIR Interactions Associated with Survival After HCT

The *KIR* locus contains genes that are centromeric (*Cen*) and telomeric (*Tel*). Dividing the B/x haplotype into *Cen* and *Tel* segments showed that the *B* haplotype genes in the *Cen* region had a stronger effect in improving the overall survival after transplantation than those in the *Tel* region. Specifically, individuals who were *Cen-B/B/Tel-X/X* (*KIR2DS2* and/or *KIR2DL2*, no *KIR2DL3/X*) versus *Cen-A/A/Tel-X/X* (*KIR2DL3* only/X) showed an increase in overall and disease-free survival. Again, no *KIR* effect was seen for ALL patients [87]. While promising, the proportion of *Cen-B/B* patients in the study was 11 %. When the cohort was expanded to include almost 500 additional AML patients (*N* = 1532), the association of leukemia-free survival (LFS) with donors having two or more *B* motifs was maintained. In addition to testing the B motif association, the authors sought to assess donor *KIR-HLA* recipient combinations with outcome [85]. Individuals who are *Cen-B/B* have activating *KIR* genes that encode inhibitory KIRs specific for the *C1* and *C2* epitopes of *HLA-C*. The authors examined the interaction between donor *KIR* B genes and recipient class I HLA KIR ligands and found transplants mismatched at *HLA-C1* experienced an almost twofold reduction in LFS by the *KIR B/x* donor. Interestingly this survival advantage in *C1/x* recipients compared with *C2/C2* recipients was similar irrespective of the donor KIR B status. Unlike the frequency of *Cen-B/B* in the population, approximately 85 % of the US population is *HLA C1/x*, thus making these findings very generalizable to the transplant population.

While these studies assessed *KIR* haplotypes, it has been shown that both OS and TRM are affected by individual activating KIRs in the *Tel* region, *KIR3DS1* and *KIR2DS1* [89••, 93], and that like the motifs, these individual *KIR* may interact with recipient *HLA*. Venstrom et al. [89••] found that donor *KIR2DS1* positive with *HLA-C1/x* conferred increased survival benefits (similar in effect size seen in *Cen-B/B* with *HLA C/x*) while *KIR3DS1* impacted survival but donor HLA-C match or mismatch had no enhanced effect. Thus, it appears that both independent and joint donor and recipient genetics impact survival, making it particularly important to better understand this complex interplay in large comprehensive studies.

### Selecting a Favorable KIR Donor in Unrelated HCT for AML

A prospective trial is currently accruing to test and validate the efficacy of choosing an URD for HCT based on *KIR* genotyping. Up to 600 AML patients will be enrolled to determine whether prospective selection of URDs based on a favorable *KIR* donor will reduce the cumulative incidence of relapse and improve LFS and overall survival. Preferred *KIR* donors are selected on the basis of a B content score. The KIR B–content score for each donor’s KIR genotype is defined as the number of centromeric and telomeric gene-content motifs containing B haplotype–defining genes and is classed as “Best”, “Better” or “Neutral”. This trial is not considering HLA-C/x donor variation. Once completed, this trial will provide the first prospective evidence on the utility of non-*HLA* genotypes to improve survival after URD-HCT for AML.

## Are We Ready for Primetime?

To understand where these genetic studies have led the transplant field, we consider this research in a useful framework for characterizing the spectrum of translational research [12••]. It is important to not think of these phases as linear but rather as on a continuum, accessible at any point on the path to identifying, quantifying, and characterizing the relationship of genomics with survival after HCT. Four phases (T1–T4) comprise the framework, with T0 representing the discovery of new variant associations and other biomarkers of outcome following HCT. In the T1 phase, results from T0 are moved towards the implementation of interventions or diagnostic tests, e.g., evaluation of the function of genomic variants and analyses of gene-exposure interactions. T2 research is the clinical intervention used to determine if the proposed application of T1 findings is better than the standard of care. The *KIR* genomics studies began as T0 and moved into T1, and the trial is at present in T2. T3 assesses how to implement and integrate T2, e.g., should *KIR-HLA* matching improve overall survival, we must determine how this can be implemented into clinical practice at transplant centers nationwide. Lastly, the population health impact of implementation is evaluated in T4, e.g., what the overall reduction is in death attributable to the introduction of *KIR* matching in HCT.

Most of genomic variation research related to HCT survival is at the T0 and maybe T1 level; however, it is important to realize that while these are the first steps to translating results to routine clinical practice, it is also imperative researchers are simultaneously working with the fields of health economics, comparative effectiveness, disparities, and bioethics in an effort to understand and reduce barriers that will prevent the successful application of non-HLA genomics to transplant [63]. One example of opportunities in this arena rests with the scientists working in the field of statistical genetics. High-resolution typing is expensive and time consuming. To combat this time and cost issue, for the last decade, researchers have focused on the development of statistical methods to impute HLA regions with a high degree of accuracy [94]. In the European-American population for classical HLA class I and class II genes, there is approximately 97 % accuracy [95], and these methods are rapidly expanding to include other races and ethnic groups [96, 97]. There are a number of translational research opportunities deriving from this T1 phase work, and with the accessibility of sequencing increasing, this work may move quickly through T2, as precision can be shown with studies in large sample sizes, and on to T3 and T4, implementation, e.g., where we must consider the how, where, and how much. We should not wait to think about these problems of implementation and assessment but rather understand the contribution and value of this particular research as it is moving towards clinical validation.

### Genome-Wide Association Study of Survival After HCT

While heterogeneity and small sample sizes have plagued many of these studies, there is clear translational potential and further exploration of the contribution of non-HLA genetic variation in the recipient and/or donor to the risk of mortality after HCT is warranted. To examine this hypothesis in a well-sized and well-defined study population, we have begun a genome-wide association study (GWAS) to investigate the joint and independent genetic factors in recipients and donors contributing to death after URD-HCT, called *D*etermining the *I*nfluence of *S*usceptibility*-CO*nveying *V*ariants *R*elated to 1-*Y*ear mortality after unrelated donor *B*lood and *M*arrow *T*ransplant *(DISCOVeRY-BMT)*. The two independent cohorts used for analyses include patients diagnosed with AML, ALL, or myelodysplastic syndrome (MDS), reported to the Center for International Blood and Marrow Transplant Research (CIBMTR) with a banked National Marrow Donor Program (NMDP) biorepository sample available for both the recipient and donor. Cohorts 1 and 2 include 2609 10/10 HLA-matched, T cell-replete, URD-HCT recipients treated from 2000 to 2008 and 923 8/8 HLA-matched, T cell-replete, URD-HCT recipients treated from 2000 to 2011, respectively. Of the 2609 patients, 1116 (43 %) died before 1-year post-transplant, and of the 923 patients, 368 (40 %) died before 1-year post-HCT.

### Competing Risk Models in GWAS

To analyze TRM, we must consider that TRM and DRM are competing risks for the outcome of death [98•]. Broadly, two approaches can be used to model competing risks: cause-specific hazard functions, constructed using a Cox proportional hazard model, defining causes other than the one we are interested in as censored failures, or we can model the cumulative incidence functions of the different event types using a subdistribution hazard model which takes into account competing risks rather than merely censoring them [99]. The modeling of the cause-specific hazards is appropriate when the goal is to assess if a factor is associated with the risk of a specific cause of failure. However, when the goal is to compare the observed incidence of events from a given cause between groups, the subdistribution hazard should be used. Because the effect of a covariate on cause-specific hazard function for a particular cause can be different from its effect on the subdistribution function of the corresponding cause, the cause-specific hazard function and subdistribution can give different results [100, 101]. These two analyses both provide important information, and it is recommended both methods are used when measuring associations with competing risk [102]. However, of the studies reviewed (Table 1), more often than not only cause-specific hazard was done and not both. This inconsistency in approach can be remedied going forward by leveraging both methods to better understand the contribution of genetic variation to survival after HCT.

To date, competing risk events have not been analyzed on a genome-wide scale and are often thought of as unique to the HCT field. However, as cancer treatments improve and more follow-up data are available, being able to analyze rare and common variation using competing risk analyses will be necessary in order to assess the role of genetic variation in competing clinical outcomes. At present, existing software scaled for millions of analyses is not available to test competing risk events; therefore, we are in the process of building custom R statistical packages to analyze both common and rare genetic variations in this setting.

### DISCOVeRY-BMT Study Design and Power

**Fig. 1:**
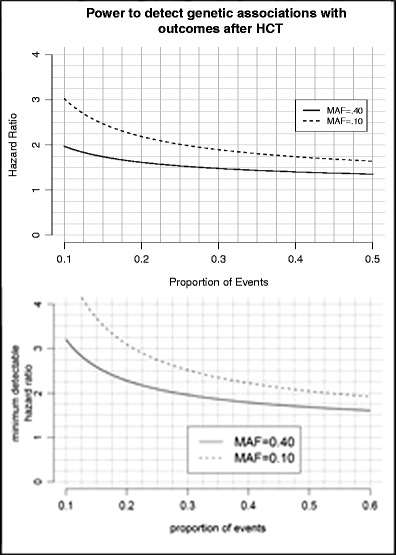
Power to detect associations with DRM, TRM, and TRM subtypes is shown in the Fig. 1. The *x-axis*, showing the proportion of events, can be used to determine power for a range of survival outcomes following HCT from 10 to 50 % in frequency. The *dashed* and *solid lines* reflect minor allele frequencies of 0.10 and 0.40, respectively. Thus, for example, given a survival outcome occurring in 25 % of DISCOVeRY-BMT cohort 1 and a minor allele frequency of 0.40, we have power to detect hazard ratios of approximately 1.5. A lower MAF of 0.10 yields power to detect hazard ratios of 2.0

The study design is illustrated in Table 2. We present some example power calculations for assessing the hazard of TRM overall as well as gene-drug interactions under a competing risk  hazard model. Given that the minimum number of TRM subtype deaths (death due to GvHD, infection, and organ failure) and overall TRM is between 10 % and 40 % and assuming a minor allele frequency (MAF) of 0.40, we have excellent power to detect hazard ratios between 1.98 and 1.38, respectively (Fig. 1). Considering a reduced population comprising only patients given myeloablative cyclophosphamide (either TBI or Bu), approximately 70 % of all patients in the first cohort, and assuming 40 % of URD-HCT recipients experience TRM before 1-year post-transplant, we will have power to detect hazard ratios from 1.75 to 2.25 for MAF varying between 40 % to 10 %, respectively. Unfortunately, in contrast to the relative homogeneity of myeloablative conditioning regimens used in URD-HCT (BuCY or CyTBI), reduced intensity/non-myeloablative conditioning regimens are heterogeneous in both drugs and doses therefore precluding a well-powered GWAS. DISCOVeRY-BMT is a large homogeneous population that will be able to unambiguously identify clinically relevant hazards attributable to the joint and/or independent effects of genetic variation. Thus, it is the first step towards translating these data clinically and defining the biology of these devastating and poorly understood endpoints [12••].

**Table 2:** DISCOVeRY-BMT study design

Genetic variation	Study population	Exposure	Survival outcomes: overall, TRM and DRM
Common (Illumina Omni Express chip + imputation) and rare (Illumina Exome chip) genetic variation	Donor and recipient pairs in two cohorts (C1 = 2601, C2 = 923)	–	AIM 1
		Non-myeloablative therapy versus myeloablative therapy	AIM 2
		Cyclophosphamide	AIM 3
		+Total body irradiation versus	
		+busulfan	

The DISCOVeRY BMT study design includes analyses of common and rare genetic variation in two cohorts. Specifically, we are undertaking a GWAS to map the independent and joint effects of recipient and donor genetic variation associated with survival outcomes after HLA-matched unrelated donor BMT in thousands of donor-recipient pairs. The purpose of specific aims 2 and 3 are to determine if conditioning regimens modify associations between recipient and/or donor genetic variants and TRM in the same population as aim 1

## Conclusion

The rapidly growing number of URD HCTs coupled with a high TRM yields an increasingly significant public health problem. Given TRM is the limiting factor to referring more potential patients and extending survival in existing patients, it is a clear target for translational research to increase the success and utilization of HCT as a curative therapy. So far, candidate gene studies have pursued discovery studies of genetic variation with the intent of making them clinically actionable but have had limited success in replication and thus are stuck in the T0 phase. When considering the KIR-HLA findings and the subsequent trial, we describe this as T2 research, with the understanding that the implementation (T3) and assessment of the impact (T4) will follow. Should this be successful, it will be the first demonstration that better matching using non-HLA alleles is not only possible but can also actively reduce death rates.

DISCOVeRY-BMT is also designed to identify polymorphisms in recipients and/or unrelated donors with the potential for better recipient-donor pairing, the identification of patients at risk for TRM, or a high probability of DRM (T1 phase) with the intent of moving through the continuum of translational research. Importantly, the data generated from this project will be made publicly available to provide a very unique resource of a highly specialized, curative therapy where many additional hypotheses can be tested or confirmed.
